# Distinct Lineage of Slow-Cycling Cells Amidst the Prevailing Heterogeneity in Glioblastoma

**DOI:** 10.3390/cancers15153843

**Published:** 2023-07-28

**Authors:** Sukrit Mahajan, Mirko H. H. Schmidt

**Affiliations:** Institute of Anatomy, Medical Faculty Carl Gustav Carus, Technische Universität Dresden School of Medicine, Fetscherstr 74, 01307 Dresden, Germany

Glioblastoma (GBM) is the most aggressive form of primary brain tumor in adults. It has hallmarks of rapid growth, invasiveness, extensive angiogenesis, and immune evasion [1]. The standard care and treatment for GBM patients involves surgical resection, radiotherapy, and chemotherapy; however, the patient prognosis is poor, with a median survival of 15 months [2]. In fact, most patients succumb to tumor relapse, which has been attributed to a subset of cancer cells called cancer stem cells (CSCs) [3].

CSCs are capable of self-renewal and differentiation and are endowed with the capacity to produce differentiated progenies that constitute the bulk of the tumor mass [4]. The concept of CSCs being involved in tumor initiation has been proposed in various malignancies, including glioblastoma. These cells comprise a heterogeneous population with multiple factors, both intrinsic and extrinsic, contributing to their phenotypic variation [5,6,7]. The survival of these cells post glioma therapy has been a leading cause for the failure of various treatment regimens. In recent years, a subpopulation of CSCs showing a reduced frequency of cell cycling has been gaining attention. With growing evidence from mouse models, xenograft outgrowth assays and scRNAseq analysis, these cells, called slow-cycling cells (SCCs), seem to play a role in glioma development and resistance to treatment [8,9,10]. Interestingly, due to the heterogeneous nature of GBM, lineage-tracing assays have been carried out to determine distinct tumor cell populations within the glioma mass [8]. However, whether or not SCCs give rise to a unique population in the glioma mass with a distinct lineage remains to be studied.

In this regard, the study by Yang et al. [11] aims to characterize the lineage of slow-cycling cells involved in GBM, and in doing so address important questions regarding tumor heterogeneity in gliomas from the perspective of CSCs. Previously, the authors have published work on SCCs [9,12] wherein they identified SCCs in gliomas and observed them to be highly infiltrative, resistant to treatment and, most importantly, capable of initiating tumor formation, similar to CSCs. In the current study, the authors follow up on their previous work. They draw a comparison between SCCs and a diverse population of GBM cells expressing stem-cell like markers, namely CD133, CD44, ITGB8, PTPRZ1 and SOX2 [13,14,15,16,17,18]. In doing so, they aim to delineate whether SCCs show any kind of overlap with other cells known to constitute the glioma CSC population. The authors chose these markers since they are overexpressed in CSCs and have been used in various studies for isolating CSCs from the tumor mass and performing further analysis [19,20,21,22].

The authors began by investigating the expression of different CSC markers (mentioned above) in SCCs isolated from patient-derived GBM cells. With the help of flow cytometry and bulk RNA sequencing, they found that SCCs showcase varying levels of expression in different CSC markers.

Following this, Yang et al. compared SCCs with fast-cycling cells (FCCs) and other cells expressing CSC markers, using scRNAseq. The authors had previously identified SCCs through a specific lipid metabolism signature that was elevated compared to the other cell populations [9]. They used the same signature to differentiate the SCCs from the other cell types in the scRNAseq screen. It was observed that SCCs had significant transcriptomic differences compared with other cell populations. Furthermore, a limited cellular overlap was observed between SCCs and other cell populations, indicating that SCCs constitute a unique population in the glioma mass.

On observing the limited overlap between the different cellular entities, the authors went on to perform a trajectory analysis of the gene expression changes to uncover any relationships that these cell populations might have with each other. The phylogenetic tree produced from the trajectory analysis placed SCCs at one end of the phylogenetic tree, closer to the CD44^high^ population, and all other populations (CD133^high^, ITGB8^high^, PTPRZ1^high^, SOX2^high^) at the other end of the tree, with CD133^high^ cells being placed at the farthest end. This provided a clear distinction of the cell lineage between SCCs and CD133^high^ cells. Furthermore, the authors went on to determine the effects of high and low expressions of different markers in patient survival. For this, they performed a functional analysis of the different populations to assess their relationship with the diseased state. Interestingly, high SCC scores and high CD44 expression led to decreased survival in patients, on analyzing the TCGA dataset and comparing to cells with low marker expression.

Moreover, the authors compared cell populations expressing dual CSC markers (instead of the single marker in each case) with SCCs, wherein they observed no cellular overlap and a high difference at the transcription level. Further, on analyzing the survival data of patients with different cancer cell populations, the authors went on to study the sensitivity of these cell populations against temozolomide (TMZ). For this, they tested the SCC population and the CD133^high^ cells, which were isolated from the same primary GBM patient line. They observed that SCCs were more resistant to TMZ treatment compared to CD133^high^ cells.

Thus, the authors provide an insight into the heterogeneous nature of gliomas through the perspective of a distinct population of CSCs. The characterization of different CSC populations using expression markers, cell cycle profiling and sensitivity to TMZ treatment showcases the heterogeneity in GBM, ranging from the molecular to the cellular. An important element in the study is the signature used for distinguishing SCCs from the rest of the CSC population. SCCs show an elevated level of lipid metabolism and even autophagy [9], and these pathways have been found to be employed by tumor cells for resisting chemotherapy in different types of cancer [23,24,25,26,27]. Thus, targeting these pathways in the context of GBM might prove helpful in disrupting the heterogeneity in GBM.

In conclusion, SCCs are proving to be a critical population in GBM heterogeneity. Thus, targeting multiple stemness markers present on CSCs by using combinatorial therapies might be very beneficial in treating GBM.
